# Stable porphyrin Zr and Hf metal–organic frameworks featuring 2.5 nm cages: high surface areas, SCSC transformations and catalyses†
†Electronic supplementary information (ESI) available: General experimental, syntheses and characterization of the complexes mentioned in the manuscript, details of the single crystal diffraction experiments, PXRD, TG and additional figures. CCDC 1043280, 1043281, 1043914 and 1043915. For ESI and crystallographic data in CIF or other electronic format see DOI: 10.1039/c5sc00213c



**DOI:** 10.1039/c5sc00213c

**Published:** 2015-03-31

**Authors:** Jun Zheng, Mingyan Wu, Feilong Jiang, Weiping Su, Maochun Hong

**Affiliations:** a State Key Laboratory of Structure Chemistry , Fujian Institute of Research on the Structure of Matter , Chinese Academy of Sciences , Fuzhou , Fujian 350002 , China . Email: wumy@fjirsm.ac.cn ; Email: wpsu@fjirsm.ac.cn; b University of Chinese Academy of Sciences , Beijing , 100049 , China

## Abstract

Two isostructural porphyrin Zr and Hf metal–organic frameworks (**FJI-H6** and **FJI-H7**) are rationally synthesized, and are constructed from 2.5 nm cubic cages. Notably, they both possess high water and chemical stability and can undergo single-crystal to single-crystal transformations to embed Cu^2+^ ions into the open porphyrin rings. **FJI-H6** has a high BET surface area of 5033 m^2^ g^–1^. Additionally, they exhibit promising catalytic abilities to convert CO_2_ and epoxides into cyclic carbonates at ambient conditions.

## Abstract 2

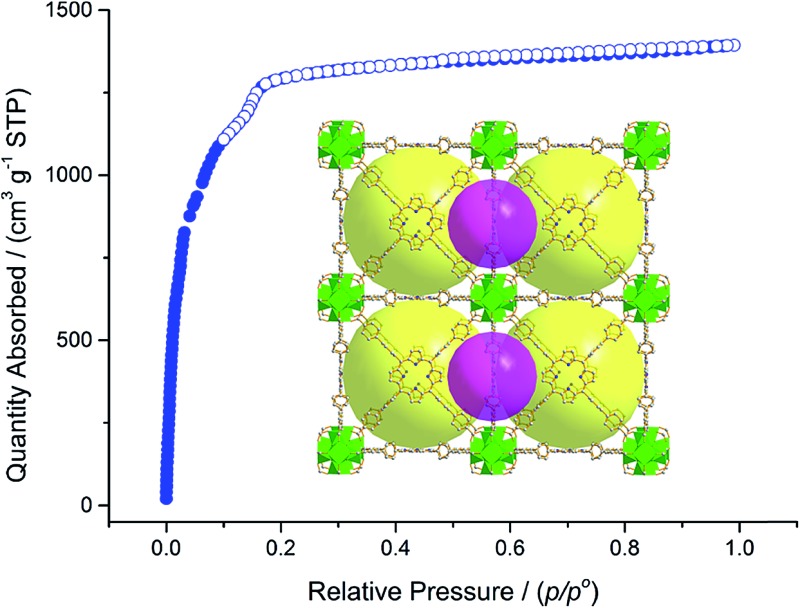
Two isostructural porphyrin Zr and Hf metal–organic frameworks (**FJI-H6** and **FJI-H7**) are rationally synthesized, and are constructed from 2.5 nm cubic cages.

## 

Owing to their high surface area, permanent porosity and tunable pores, metal–organic frameworks (MOFs) have been used for catalysis,^1^ gas separation and storage,^2^ and drug delivery.^3^ Nonetheless, one big obstacle to the practical applications of MOFs is their stability, which includes water and chemical stability. To target stable MOFs, one effective method is the use of high-valence metal ions such as Fe^3+^, Al^3+^, Cr^3+^, Zr^4+^ and Hf^4+^ ions as the metal nodes.^4^ Compared with the traditional Cu^2+^, Zn^2+^ and Co^2+^ ions,^5^ the aforementioned trivalent or tetravalent metal ions will form stronger bonds with carboxylate groups according to the theory of hard and soft acids and bases. Therefore, the stability of the obtained frameworks will be strengthened. Furthermore, these high-valence metal ions tend to form highly connected inorganic clusters *via* the OH^–^ and/or O^2–^ bridges, which also significantly contributes to the stability of the frameworks. As for the Zr^4+^ ion, it prefers to form the classical 12-connected Zr_6_O_4_(OH)_4_ node. When assembled with linear carboxylate ligands, 3D fcu frameworks with ordered cubic cages (known as the UiO series) can be obtained.^6^ These kinds of Zr-based MOF often have high water and chemical stability, and can even serve as water adsorbents.^7^ Up to now, extensive investigation has been carried out to tune the porosity of the UiO series of Zr-MOFs by selectively removing organic linkers^8^ or by functionalizing the ligands (by pre-modification or post-synthetic methods).^9^ However, there are several examples based on the assembly of Zr_6_O_4_(OH)_4_ clusters with polycarboxylic ligands such as planar tetracarboxylic acids.^7b,10^ In addition, compared with Zr-based MOFs, Hf-based MOFs are also rare.^6f,11^ Herein, we present two ultra-stable metal–organic frameworks ([Zr_6_O_4_(OH)_4_(H_2_TBPP)_3_]_*n*_·(solvent)_*x*_) (**FJI-H6**) and ([Hf_6_O_4_(OH)_4_(H_2_TBPP)_3_]_*n*_·(solvent)_*x*_) (**FJI-H7**), which are isostructural and both constructed from M_6_O_4_(OH)_4_(CO_2_)_12_ nodes (M = Zr, Hf) and porphyrin tetracarboxylic ligands (H_6_TBPP = 4′,4′′′,4′′′′′,4′′′′′′′-(porphyrin-5,10,15,20-tetrayl)tetrakis([1,1′-biphenyl]-4-carboxylic acid)). As expected, both **FJI-H6** and **FJI-H7** have high water and chemical stability and can undergo a single-crystal-to-single-crystal (SCSC) transformation to embed Cu^2+^ ions into the open porphyrin rings. Interestingly, they both feature 2.5 nm cages. Notably, **FJI-H6** has a high BET surface area of 5033 m^2^ g^–1^.

## Results and discussion

### Syntheses and structures of porphyrin Zr and Hf MOFs

**Fig. 1:**
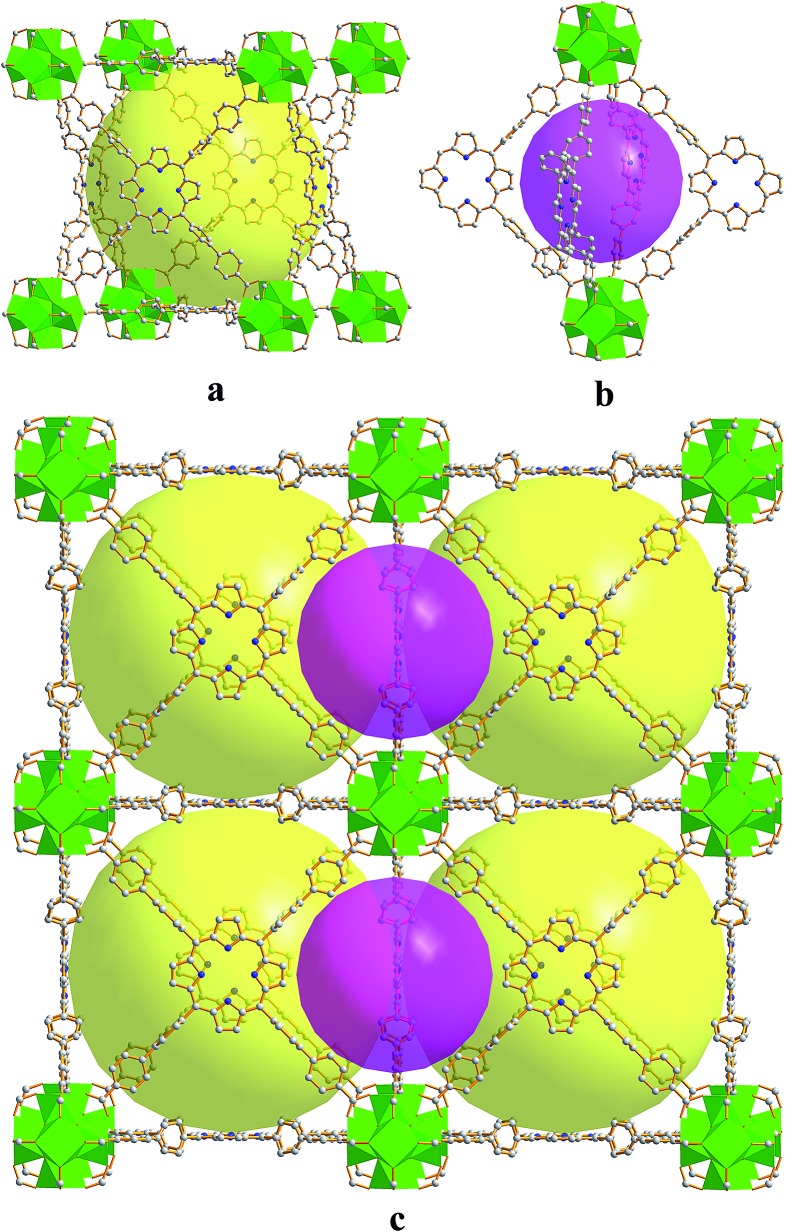
(a) The large cubic cage constructed from six porphyrin ligands and eight Zr_6_O_4_(OH)_4_ clusters. (b) The small octahedral cage constructed from four porphyrin ligands and two Zr_6_O_4_(OH)_4_ clusters. (c) Packing of the two kinds of cages.

Reaction of H_6_TBPP with ZrCl_4_ or HfCl_4_ modulated by benzoic acid gives rise to dark red crystals of **FJI-H6** or **FJI-H7**. Single crystal X-ray structural analysis shows that **FJI-H6** and **FJI-H7** are isostructural.^12^ Therefore, we chose **FJI-H6** as the example in the following discussion. **FJI-H6** crystallizes in the high symmetry space group *Pm*3*m*. In the Zr_6_O_4_(OH)_4_ cluster, six equivalent Zr^4+^ ions are in a square-antiprismatic O_8_ coordination environment and form a regular octahedron. In this Zr_6_ octahedron, the eight triangular faces are alternatively capped by four μ_3_-OH^–^ and four μ_3_-O^2–^ groups. Additionally, the twelve edges of the Zr_6_ octahedron are bridged by twelve carboxylate groups from twelve unique H_2_TBPP ligands. At the same time, each H_2_TBPP ligand, in which the peripheral four phenyl rings are coplanar with the inner porphyrin rings, links four independent Zr_6_O_4_(OH)_4_ clusters. Thus, a rarely seen (4,12)-connected ftw framework can be acquired.^10a,11b,13^ **FJI-H6** has two kinds of polyhedral cages, *i.e.* a small octahedral cage and a large cubic cage. As seen in Fig. 1, the octahedral cage is constructed from two Zr_6_O_4_(OH)_4_ clusters and four H_2_TBPP ligands, with a cavity diameter of *ca.* 1.5 nm. However, the cubic cage consists of eight Zr_6_O_4_(OH)_4_ clusters as the vertices and six H_2_TBPP ligands as the sides. Significantly, the diameter of the cubic cage is approximately 2.5 nm, which is larger than that in PCN-221 (2.0 nm).^11*b*^ Accordingly, the available volume is 15 000 Å^3^. Additionally, the window of the cubic cage is 1.2 nm × 2.0 nm, which allows large organic molecules to freely get in and out.

### Gas adsorption experiments and stability tests

**Fig. 2:**
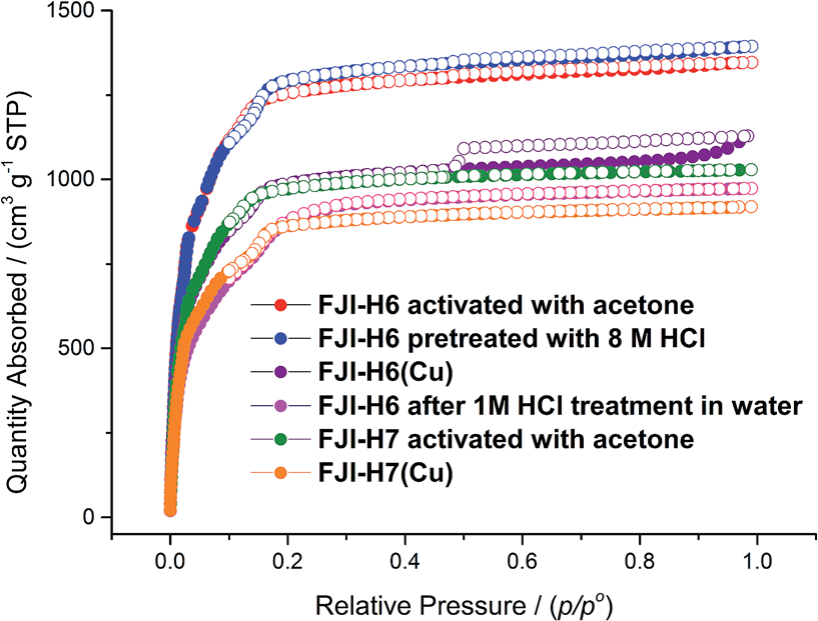
Experimental N_2_ adsorption isotherms for **FJI-H6**, **FJI-H6(Cu)**, **FJI-H7** and **FJI-H7(Cu)**.

Calculation by PLATON software^14^ reveals that in **FJI-H6** the free volume is up to 78.6%. For **FJI-H6** the permanent porosity is confirmed by an N_2_ adsorption isotherm measured at 77 K. The sample exchanged with acetone exhibits a reversible type I isotherm and has a saturated uptake of 1346 cm^3^ g^–1^ at 1 atm (Fig. 2). When pre-treated with 8 M HCl, the value of the N_2_ adsorption slightly increases to 1393 cm^3^ g^–1^, which indicates that **FJI-H6** is stable with respect to the acid. From the above data, the calculated BET surface area of the sample exchanged with acetone is up to 5007 m^2^ g^–1^ (5033 m^2^ g^–1^ for the sample pre-treated with 8 M HCl), which is much larger than those of PCN-222(Fe) (2200 m^2^ g^–1^),^4*b*^ NU-1000 (2320 m^2^ g^–1^),^15^ PCN-223(Fe) (1600 m^2^ g^–1^),^10*c*^ PCN-94 (3377 m^2^ g^–1^),^10*b*^ NU-1100 (4020 m^2^ g^–1^)^10*a*^ and PCN-229 (4619 m^2^ g^–1^),^13^ but less than those of the just reported NU-1103 (5646 m^2^ g^–1^) and NU-1104 (5290 m^2^ g^–1^).^10*e*^ In addition, **FJI-H6** also has a high total pore volume of 2.16 cm^3^ g^–1^. The experimental BET surface area and pore volume are consistent with theoretical values calculated by Poreblazer^16^ (accessible surface area: 4695 m^2^ g^–1^; pore volume: 2.06 cm^3^ g^–1^),^14^ which demonstrates that the sample is fully activated. Additionally, **FJI-H6** also shows good capacity for H_2_ storage. The H_2_ uptake reaches 172 cm^3^ g^–1^ (1.54 wt%) at 1 atm and 77 K, and 108 cm^3^ g^–1^ (0.94 wt%) at 87 K and 1 atm. Moreover, the adsorption heat of H_2_ calculated by the Clausius–Clapeyron equation is 6.54 kJ mol^–1^ at zero coverage and decreases slowly with increasing H_2_ loading. These values are comparable to those of famous MOF materials, such as HKUST-1 (6.6 kJ mol^–1^),^17^ MOF-5 (5.2 kJ mol^–1^),^17^ and NOTT-122 (6.0 kJ mol^–1^).^18^ As for **FJI-H7** the sample exchanged with acetone also exhibits a reversible type I isotherm and has a saturated uptake of 1029 cm^3^ g^–1^ at 1 atm. From the above data, the calculated BET surface area of **FJI-H7** is up to 3831 m^2^ g^–1^, which is among the highest reported for Hf-based MOFs.^11^

Since the Zr_6_O_4_(OH)_4_ and Hf_6_O_4_(OH)_4_ clusters are both highly connected with twelve carboxylate groups, **FJI-H6** and **FJI-H7** are expected to have a high stability. To test their stabilities, we immersed a microcrystalline sample of **FJI-H6** or **FJI-H7** into water with various pH values for 24 h (see Fig. S2†). The PXRD patterns of the resulting samples match well with the simulated ones, which suggests that **FJI-H6** and **FJI-H7** retain their crystallinity. In particular, **FJI-H6** has high stability in acid. As seen in Fig. 2, the curves and the adsorption values for the sample treated with 8 M HCl do not deviate much from those of the untreated sample.

### Incorporating Cu^2+^ ions into the open porphyrin rings *via* SCSC transformations

Considering that in both **FJI-H6** and **FJI-H7** two nitrogen atoms of the porphyrin ring are not deprotonated, we attempted to introduce a second kind of metal ion into the framework. Immersing single-crystals of **FJI-H6** or **FJI-H7** into a solution of 0.5 M Cu(NO_3_)_2_ in *N*,*N*-dimethyl formamide (DMF) at 85 °C for 72 h results in metallated **FJI-H6(Cu)** or **FJI-H7(Cu)**. As anticipated, single crystal X-ray structural analysis definitively shows that in both **FJI-H6** and **FJI-H7** the Cu^2+^ ions have been embedded in the porphyrin rings. The Cu^2+^ ion is in a square planar N4 coordination environment with two axial sites exposed, which is typical for divalent metal ions in metal–porphyrin complexes.^19^ We believe that, although there are several examples of exchanging metal ions in porphyrin MOFs, this is the first observation of incorporating metal ions into open porphyrin rings *via* SCSC transformations in porphyrin MOFs. N_2_ adsorption measurements at 77 K for **FJI-H6(Cu)** also show a type I isotherm. At 1 atm, **FJI-H6(Cu)** has the maximum N_2_ adsorption of 1128 cm^3^ g^–1^, which is smaller than that of **FJI-H6**. Accordingly, the BET surface area of **FJI-H6(Cu)** is reduced to 3731 m^2^ g^–1^. Similarly, the maximum N_2_ adsorption and BET surface area of **FJI-H7(Cu)** (918 cm^3^ g^–1^ and 3195 m^2^ g^–1^, respectively) are also lower than those of **FJI-H7**. The reason may be ascribed to the introduction of Cu^2+^ ions, which can slightly diminish the surface area.

### Cycloaddition reactions of CO_2_ with epoxides

**Scheme 1:**
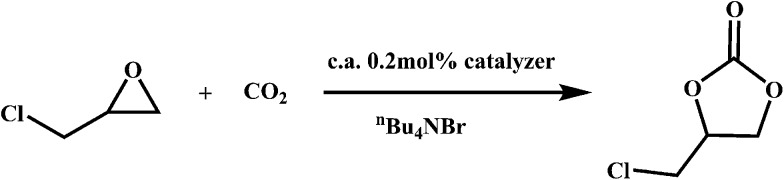
Cycloaddition reactions of CO_2_ with epoxide catalyzed by **FJI-H6(Cu)**, **FJI-H7** and **FJI-H7(Cu)**.

Recently, owing to global warming, efficient CO_2_ capture and storage is urgently needed to reduce CO_2_ emissions before scientists find a practical clean energy. If we can convert this abundant inorganic waste into usable organic chemicals utilizing reasonable reactions at ambient conditions, the above problem can be perfectly solved. One practical method is the synthesis of cyclic carbonates from CO_2_ and epoxides, which have extensive applications as degreasers, polar aprotic solvents and electrolytes in lithium ion batteries. Though many catalysts have been explored for the above reaction, metalloporphyrins show relatively high catalytic activity.^20^ Hence, we have evaluated **FJI-H6**, **FJI-H6(Cu)**, **FJI-H7** and **FJI-H7(Cu)** as heterogeneous catalysts for the cycloaddition reaction of CO_2_ with epoxides (Scheme 1). Typically, 25.5 mmol 3-chloropropylene oxide, 0.51 mmol (2.0 mol%) tetrabutylammonium bromide and 0.051 mmol (0.2 mol%) catalyst were added to a thick-walled glass tube with a stirring bar. The tube was placed under vacuum and then purged with CO_2_. The above cycle was repeated three times. Finally, the pressure of CO_2_ was set as 1 atm. The mixture was stirred at 25 °C for 60 hours. Analysis of the resulting solution by gas chromatography indicated that 52.6%, 61.8%, 64% and 66.5% of the epoxide was converted into the cyclic carbonate for **FJI-H6**, **FJI-H6(Cu)**, **FJI-H7** and **FJI-H7(Cu)**, respectively. Though the yields are not very high compared with homogeneous catalysts, it is nevertheless promising considering the low temperature and pressure. Compared with **FJI-H6** or **FJI-H7**, **FJI-H6(Cu)** or **FJI-H7(Cu)** has a higher catalytic ability. The reason may be that, as a Lewis catalytic site, the embedded Cu(ii) ion in the porphyrin ring contributes to some extent. At the same time, the Hf-based MOFs **FJI-H7** and **FJI-H7(Cu)** have higher catalytic abilities than the corresponding Zr-based MOFs **FJI-H6** and **FJI-H6(Cu)**, respectively since the Hf ion is more oxophilic than the Zr ion and acts as a stronger Lewis acid. Additionally, the PXRD patterns of **FJI-H6(Cu)**, **FJI-H7** and **FJI-H7(Cu)** after catalyses are in good agreement with the simulated ones (see Fig. S2†), which further demonstrates that they all retain their framework. However, it is a pity that **FJI-H6** lost its crystallinity during the catalytic process.

## Conclusions

In conclusion, we report the design and synthesis of two ultra-stable MOFs **FJI-H6** and **FJI-H7**, which both feature 2.5 nm cages. In particular, **FJI-H6** has a high BET surface area of 5033 m^2^ g^–1^. Due to the high connectivity of the M_6_O_4_(OH)_4_ clusters (M = Zr and Hf), **FJI-H6** and **FJI-H7** are stable in water with pH values ranging from 0 to 10. Interestingly, they can undergo a single-crystal to single-crystal transformation to embed Cu^2+^ ions into the porphyrin rings, which also indicates their high chemical stability. Additionally, preliminary catalysis evaluation shows that **FJI-H6(Cu)**, **FJI-H7** and **FJI-H7(Cu)** exhibit promising catalytic capacity for converting CO_2_ and epoxides into cyclic carbonates at low temperature and pressure. Consequently, **FJI-H6**, **FJI-H7** and their derivatives may be applied in catalysis due to their high surface area, ultra-high stability and easy post-modification. Further research is ongoing.
