# Supplementary material for: Stable porphyrin Zr and Hf metal–organic frameworks featuring 2.5 nm cages: high surface areas, SCSC transformations and catalyses
Source: Chem Sci. 2015 Mar 31;6(6):3466–70. doi: 10.1039/c5sc00213c (PMC5492870; doi:10.1039/c5sc00213c)
Supplement: Supplementary file 1 [file SC-006-C5SC00213C-s001.pdf]

Electronic Supplementary Material (ESI) for Chemical Science.
This journal is © The Royal Society of Chemistry 2015

Stable porphyrin Zr and Hf metal-organic frameworks featuring 2.5 nm cages and high surface areas

Jun Zheng,[a,b] Mingyan Wu,*[a] Feilong Jiang,[a] Weiping Su*[a] and Maochun Hong[a]

[a]State Key Laboratory of Structure Chemistry, Fujian Institute of Research on the Structure of Matter, Chinese Academy of Sciences, Fuzhou, Fujian, 350002, China

[b] University of the Chinese Academy of Sciences, Beijing, 100049, China

Supporting Information:

Experimental Section

Fig. S1 Powder X-ray diffraction (PXRD) patterns of **FJI-H6**, **FJI-H6(Cu)**, **FJI-H7** and **FJI-H7(Cu)**.

Fig. S2 Powder X-ray diffraction (PXRD) patterns of **FJI-H6** and **FJI-H7** treated with aqueous solution with various pH values.

Fig. S3 Powder X-ray diffraction (PXRD) patterns of **FJI-H6**, **FJI-H6(Cu)**, **FJI-H7** and **FJI-H7(Cu).** after catalysis reaction

Fig. S4 TGA curves of **FJI-H6** and **FJI-H7**.

Fig. S5 H_2 adsorption isotherm of **FJI-H6** at 77 K and 87 K.

Fig. S6 Isosteric heat of H_2 adsorption at low coverage for **FJI-H6**.

Table S1 Crystal data and structure refinement for **FJI-H6**.

Table S2 Crystal data and structure refinement for **FJI-H6(Cu).**

Table S3 Crystal data and structure refinement for **FJI-H7**.

Table S4 Crystal data and structure refinement for **FJI-H7(Cu).**

Experimental Section

Synthesis of FJI-H6:

$ZrCl_4$ (24 mg) was dissolved in 8 mL of N,N-dimethylformamide (DMF) in a 25 mL pyrex vial, to which 1.0g benzoic acid was added. The mixture was heated in 85 ºC oven for 1 hours and cooled to the room temperature. Then, H_6TBPP (20 mg) was added to the above solution, which was sonicated for a while. The resulting mixture was heated in 120 ºC oven for 96 hours to yield about 14 mg of dark red crystals (yield: 38.89% based on H_6TBPP). The crystals obtained were filtered and washed with DMF. Elemental analyses calcd (%) for **FJI-H6** (After activation and absorbed small amount of water, the crystal has a formula of $[Zr_6O_4(OH)_4(H_2TBPP)_3]_n$·~20n$H_2O$): C 58.06, H 4.26, N, 3.74; found: C 57.51, H 4.17, N, 3.84

Synthesis of FJI-H6(Cu):

About 100 mg **FJI-H6** were immersed in the 0.5 mol L^{-1} $Cu(NO_3)_2$ in DMF at 85 ℃ for 72 h. During this period, the solution of $Cu(NO_3)_2$ in DMF was replaced with the fresh one about every 8 h. Then, the sample was repeated washed with DMF until the filtrate was colorless. Inductively coupled plasma (ICP) analysis shows that the ratio of Zr and Cu is *c.a.* 1:1. The reason may be that besides the Cu(II) ions incorporated in the open porphyrin rings , there are some free $Cu(NO_3)_2$ residing in the framework or some nano-size Cu_2O particles mixed in the sample. Elemental analyses calcd (%) for **FJI-H6(Cu)** after *SQUEEZED*: C 62.70, H 3.02, N, 4.06; found: C 53.35, H 3.10, N, 4.20. The large difference between the calcd and the found ones may be due to the free $Cu(NO_3)_2$ residing in the framework or some nano-size Cu_2O particles mixed in the sample, and the solvent molecules such as DMF and H_2O.

Syntheses of **FJI-H7** and **FJI-H7(Cu)** are similar to the above procedure.

X-ray Data Collection and Structure Determination of FJI-H6, FJI-H6(Cu), FJI-H7 and FJI-H7(Cu):

For the single crystal analysis of **FJI-H6**, a dark red crystal was taken directly from the mother liquor, transferred into a quartz capillary tube. The crystal was kept at 293.00(10) K during data collection on a supernova diffractometer equipped with a Multilayers mirror Cu-Kα radiation (λ = 1.5418 Å) by using a ω scan mode. The crystal structure was solved by direct method and refined by full-matrix least squares on F^2 using *SHELXTL* package. Non-hydrogen atoms were refined with anisotropic displacement parameters. The free solvent molecules are highly disordered in **FJI-H6**, and attempts to locate and refine the solvent peaks were unsuccessful. The diffused electron densities resulting from these solvent molecules were removed using the *SQUEEZE* routine of *PLATON*; structures were then refined again using the data generated. The same method was applied for **FJI-H6(Cu)**, **FJI-H7** and **FJI-H7(Cu)**. Crystal data are summarized in Table S1,Table S2, Table S3 and Table S4. Crystallographic data for the structures reported in this paper have been deposited in the Cambridge Crystallographic Data Center with CCDC reference numbers 1043280, 1043281, 1043914 and 1043915 for **FJI-H6**, **FJI-H6(Cu)**, **FJI-H7** and **FJI-H7(Cu)**.

Low Pressure Gas Sorption Measurements:

the fresh crystalline sample of **FJI-H6** was firstly exchanged in the DMF at 85 ℃ for 16 h and then acetone for three days. Then, the sample was degassed under dynamic vacuum at 80 ℃ for 10 hours. The same method was applied for **FJI-H6(Cu)**, **FJI-H7** and **FJI-H7(Cu)**. For **FJI-H6**, it can also be pretreated with 8 M HCl: *c.a.* 100 mg sample was put in a mixture of 1.5 mL 8 M HCl and 10 ml DMF at 85 ℃ for 12 h. The N_2 adsorption/desorption isotherms were measured

volumetrically using a Micromeritics ASAP 2020 surface area and pore size analyzer up to saturated pressure at 77 K. The specific surface areas were determined using the Brunauer-Emmett-Teller (BET) and the Langmuir equation from the N_2 sorption data. By applying the Clausius–Clapeyron equation to two sets of hydrogen adsorption data collected at 77K and 87K, the isosteric heat of adsorption ($\triangle H_{ads}$) can be obtained.

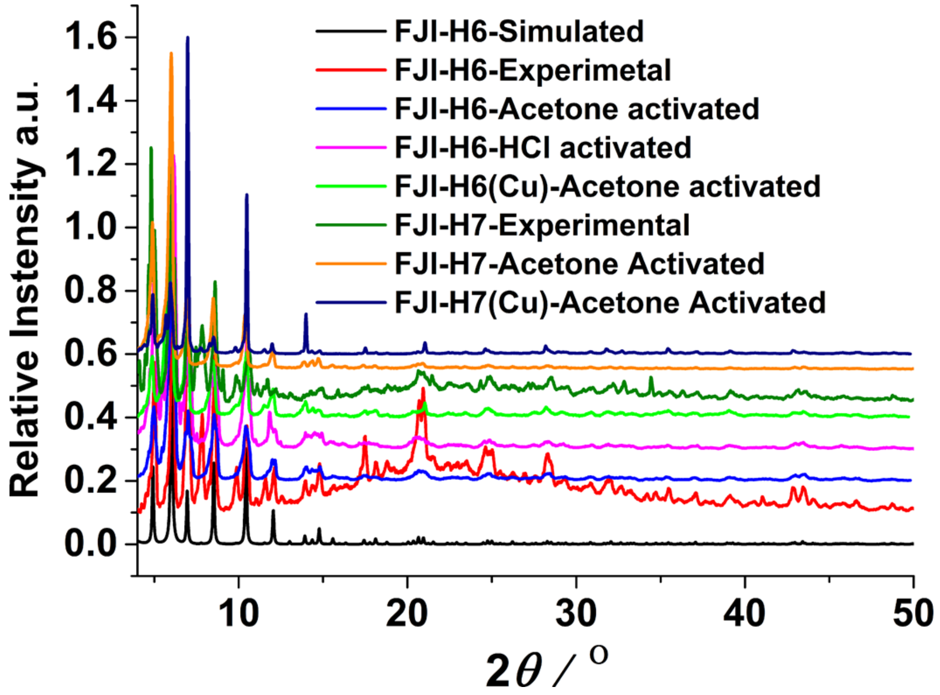

Fig. S1 Powder X-ray diffraction (PXRD) patterns of **FJI-H6**, **FJI-H6(Cu)**, **FJI-H7** and **FJI-H7(Cu).**

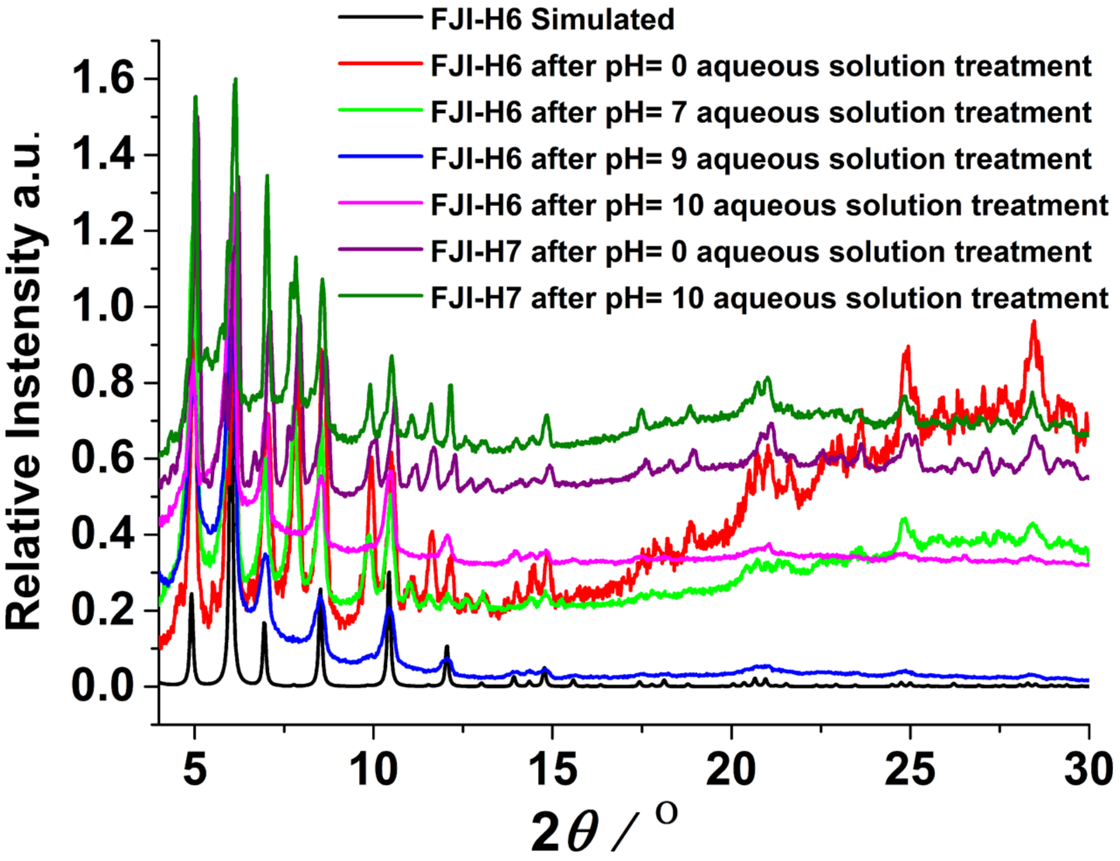

Fig. S2 Powder X-ray diffraction (PXRD) patterns of **FJI-H6** and **FJI-H7** treated with aqueous solution with various pH values.

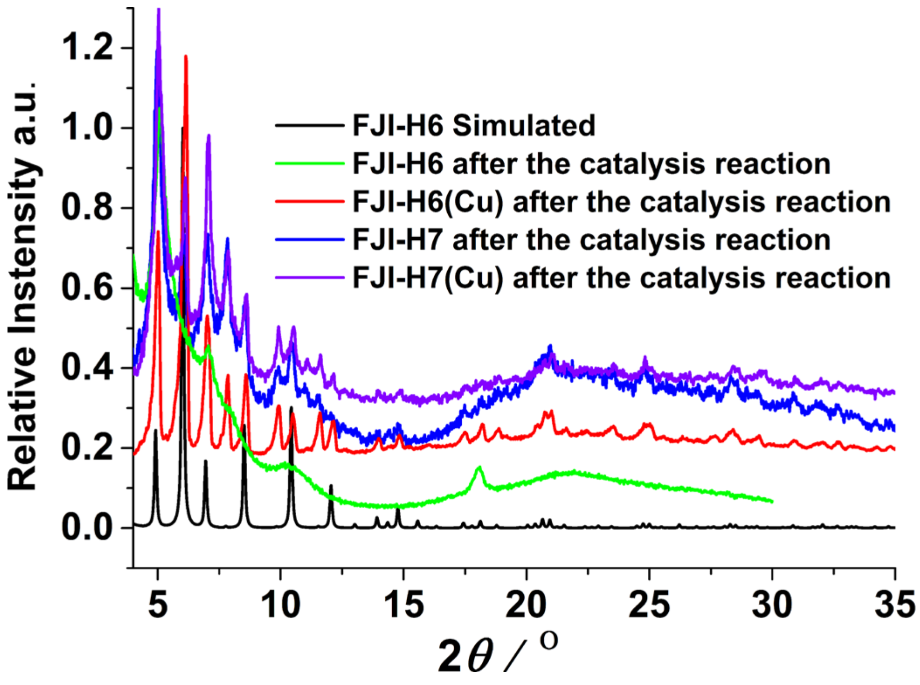

Fig. S3 Powder X-ray diffraction (PXRD) patterns of **FJI-H6**, **FJI-H6(Cu)**, **FJI-H7** and **FJI-H7(Cu).** after catalysis reaction.

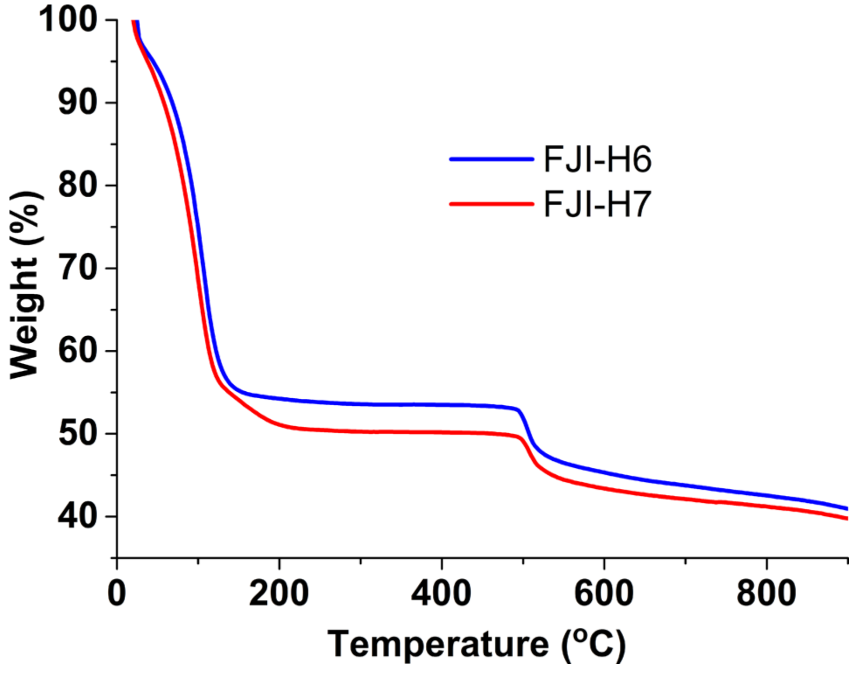

Fig. S4 TGA curves of **FJI-H6** and **FJI-H7**.

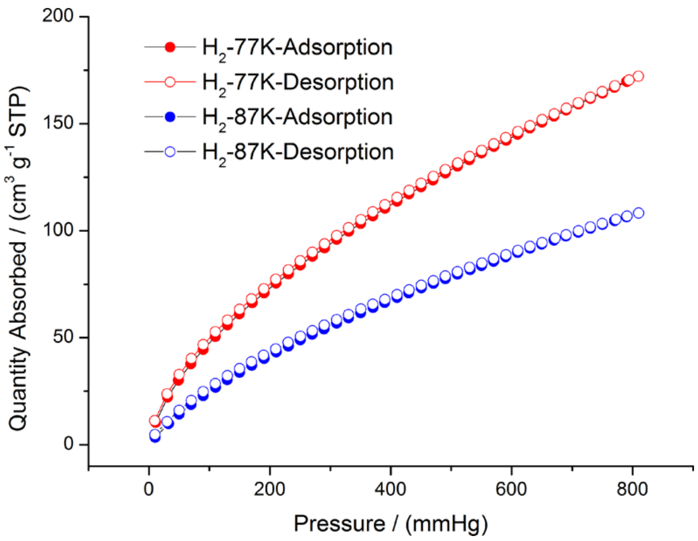

Fig. S5 H_2 adsorption isotherm of **FJI-H6** at 77 K and 87 K.

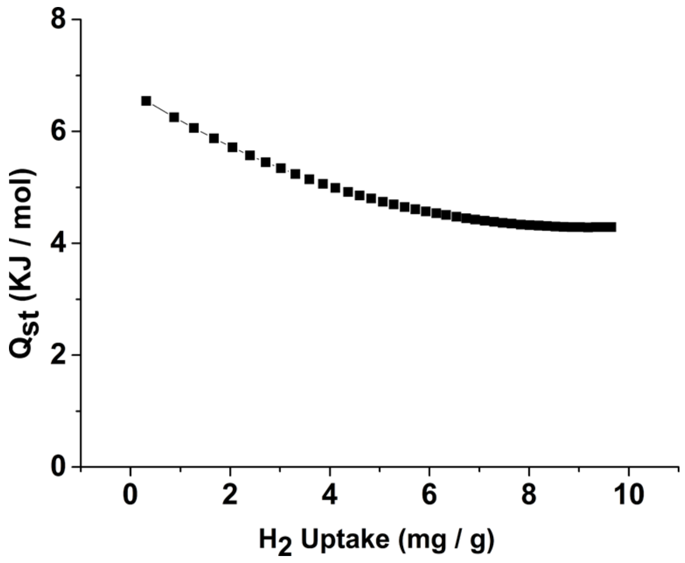

Fig. S6 Isosteric heat of H_2 adsorption at low coverage for **FJI-H6**.

Table S1. Crystal data and structure refinement for **FJI-H6**:

Identification code	**FJI-H6**
Empirical formula	$C_{216}H_{130}N_{12}O_{32}Zr_6$
Formula weight	3952.63
Temperature	293.00(10)
Wavelength	1.54184Å
Crystal system	cubic
Space group	*Pm-3m*
Unit cell dimensions.	$a = b = c = 25.43760(10)$Å
	$\alpha = \beta = \gamma = 90$ °
Volume	16459.95(19)Å^3

Z	1
Density (calculated)	0.3987 mg mm^{-3}
Absorption coefficient	0.930 mm^{-1}
F(000)	2006
Crystal size	0.12×0.12×0.12 mm
θ range for data collection.	3.8720 to 73.4480 °
Limiting indices	-13<= *h* <= 31, -30 <= *k* <= 22, -21<= *l* <= 31
Reflections collected / unique	3233 / 2798
Completeness	99.9 %
Absorption correction	multi-scan
Data/restraints/params	3233/66/83
Refinement method	Full-matrix least squares on F^2
Goodness of fit on F^2	1.065
Final *R* indices [*I*>2sigma(*I*)]	*R*1 = 0.0563, *wR*2 = 0.1595
R indices (all data)	*R*1 = 0.0596, *wR*2 = 0.1637

Table S2. Crystal data and structure refinement for **FJI-H6(Cu)**:

Identification code	**FJI-H6(Cu)**
Empirical formula	$C_{216}H_{124}N_{12}O_{32}Cu_3Zr_6$
Formula weight	4137.20
Temperature	293.00(10)
Wavelength	1.54184Å
Crystal system	cubic
Space group	*Pm-3m*
Unit cell dimensions.	$a = b = c$ = 25.4213(2)Å
	$\alpha = \beta = \gamma$ = 90 °
Volume	16428.3(4) Å^3
Z	1
Density (calculated)	0.4181 mg mm^{-3}
Absorption coefficient	1.032 mm^{-1}
F(000)	2087
Crystal size	0.09×0.09×0.09 mm
θ range for data collection.	3.888 to 73.150 °
Limiting indices	-23<= *h* <= 19, -11 <= *k* <= 30, -21<= *l* <= 31
Reflections collected / unique	3192 / 2496
Completeness	99.8 %
Absorption correction	multi-scan
Data/restraints/params	3192/54/85
Refinement method	Full-matrix least squares on F^2
Goodness of fit on F^2	1.109
Final *R* indices [*I*>2sigma(*I*)]	*R*1 = 0.0633, *wR*2 = 0.1814
R indices (all data)	*R*1 = 0.0776, *wR*2 = 0.1913

Table S3. Crystal data and structure refinement for **FJI-H7**:

Identification code	**FJI-H7**
Empirical formula	$C_{216}H_{130}N_{12}O_{32}Hf_6$
Formula weight	4476.25
Temperature	293.00(10)
Wavelength	1.54184Å
Crystal system	cubic
Space group	*Pm-3m*
Unit cell dimensions.	$a = b = c = 25.39760(10)$Å
	$\alpha = \beta = \gamma = 90$ °
Volume	16382.42(19)$Å^3$
Z	1
Density (calculated)	0.4537 mg mm^{-3}
Absorption coefficient	1.876 mm^{-1}
F(000)	2198
Crystal size	0.13×0.13×0.13 mm
θ range for data collection.	3.8770 to 73.2370 °
Limiting indices	$-21 <= h <= 31$, $-31 <= k <= 21$, $-21 <= l <= 30$
Reflections collected / unique	3164 / 2744
Completeness	99.9 %
Absorption correction	multi-scan
Data/restraints/params	3164/24/83
Refinement method	Full-matrix least squares on F^2
Goodness of fit on F^2	1.080
Final R indices [I>2sigma(I)]	$R1 = 0.0306$, $wR2 = 0.1016$
R indices (all data)	$R1 = 0.0330$, $wR2 = 0.1032$

Table S4. Crystal data and structure refinement for **FJI-H7(Cu)**:

Identification code	**FJI-H7(Cu)**
Empirical formula	$C_{216}H_{124}N_{12}O_{32}Cu_3Hf_6$
Formula weight	4660.82
Temperature	293.01(10)
Wavelength	1.54184Å
Crystal system	cubic
Space group	*Pm-3m*
Unit cell dimensions.	$a = b = c = 25.35700(10)$Å
	$\alpha = \beta = \gamma = 90$ °
Volume	16303.98(19) $Å^3$
Z	1
Density (calculated)	0.4746 mg mm^{-3}
Absorption coefficient	1.985 mm^{-1}
F(000)	2279

Crystal size	0.14×0.14×0.14mm
θ range for data collection.	3.8780 to 73.1880 °
Limiting indices	-6<= *h* <= 21, -18 <= *k* <= 26, -10<= *l* <= 31
Reflections collected / unique	3165 / 2873
Completeness	99.7 %
Absorption correction	multi-scan
Data/restraints/params	3165/30/85
Refinement method	Full-matrix least squares on F^2
Goodness of fit on F^2	1.095
Final *R* indices [*I*>2sigma(*I*)]	*R*1 = 0.0397, *wR*2 = 0.1188
R indices (all data)	*R*1 = 0.0422, *wR*2 = 0.1203
